# Supplementary material for: Toxicity, Response and Survival in Older Patients with Metastatic Melanoma Treated with Checkpoint Inhibitors
Source: Cancers (Basel). 2021 Jun 5;13(11):2826. doi: 10.3390/cancers13112826 (PMC8201158; doi:10.3390/cancers13112826)
Supplement: Supplementary file 1 [file cancers-13-02826-s001.zip › cancers-1170288-supplementary.pdf]

Supplemental files

Supplemental table S1: specific toxicities (grade ≥3) of checkpoint inhibitors per age-group

	**All patients**		**Patients aged 65-74**		**Patients aged 75 or older**		
	N	(%)*	N	(%)	N	(%)	*p-value*
**Anti-PD-1 treatment (N=920)**							
All toxicity (grade ≥3)*	140	(15.2)	67	(13.9)	73	(16.6)	*0.255*
Specific toxicity (grade ≥3)*							
Bone marrow suppression	2	(0.2)	0	(0.0)	2	(0.5)	*0.138*
Neuropathy	3	(0.3)	1	(0.2)	2	(0.5)	*0.510*
Colitis	26	(2.8)	11	(2.3)	15	(3.4)	*0.302*
Kidney function deterioration	10	(1.1)	6	(1.2)	4	(0.9)	*0.623*
Nefritis	15	(1.6)	7	(1.5)	8	(1.8)	*0.661*
Dyspnea	7	(0.8)	4	(0.8)	3	(0.7)	*0.796*
Pneumonitis	16	(1.7)	5	(1.0)	11	(2.5)	*0.089*
Adrenal gland insufficiency	3	(0.3)	3	(0.6)	0	(0.0)	*0.097*
Hypopituitary insufficiency	3	(0.3)	2	(0.4)	1	(0.2)	*0.617*
Hypo (or hyper) thyroidism	3	(0.3)	2	(0.4)	1	(0.2)	*0.617*
Fatigue	5	(0.5)	3	(0.6)	2	(0.5)	*0.729*
Rash	10	(1.1)	6	(1.2)	4	(0.9)	*0.623*
Pruritis	4	(0.4)	2	(0.4)	2	(0.5)	*0.927*
Vitiligo	4	(0.4)	2	(0.4)	2	(0.5)	*0.927*
Hepatitis	20	(2.2)	9	(1.9)	11	(2.5)	*0.510*
Other	53	(5.8)	30	(6.2)	23	(5.2)	*0.516*
**Ipilimumab**							
All toxicity (grade ≥3)*	115	(31.6)	75	(31.9)	40	(31.0)	*0.859*
Specific toxicity (grade ≥3)*							
Bone marrow suppression	2	(0.5)	1	(0.5)	1	(0.4)	*0.666*
Colitis	50	(13.7)	31	(13.2)	19	(14.7)	*0.684*

Skin toxicity	7	(1.9)	1	(0.4)	6	(4.7)	*0.005*
Adrenal gland insufficiency	12	(3.3)	8	(3.4)	4	(3.1)	*0.877*
Hypopituitary insufficiency	23	(6.3)	18	(7.7)	5	(3.9)	*0.156*
Hypo (or hyper) thyroidism	11	(3.0)	9	(3.8)	2	(1.6)	*0.224*
Hepatitis	13	(3.6)	10	(4.3)	3	(2.3)	*0.343*
Other	33	(9.1)	19	(8.1)	14	(10.9)	*0.379*
**Ipilimumab + nivolumab**							
All toxicity (grade ≥3)*	86	(42.8)	59	(41.0)	27	(47.4)	*0.409*
Specific toxicity (grade ≥3)*							
Bone marrow suppression	2	(1.0)	1	(0.7)	1	(1.8)	*0.495*
Neuropathy	2	(1.0)	2	(1.4)	0	(0.0)	*0.371*
Colitis	26	(12.9)	18	(12.5)	8	(14.0)	*0.770*
Nefritis	8	(4.0)	3	(2.1)	5	(8.8)	*0.029*
Pneumonitis	8	(4.0)	3	(2.1)	5	(8.8)	*0.029*
Adrenal gland insufficiency	6	(3.0)	3	(2.1)	3	(5.3)	*0.232*
Hypopituitary insufficiency	5	(2.5)	4	(2.8)	1	(1.8)	*0.675*
Hypo (or hyper) thyroidism	2	(1.0)	1	(0.7)	1	(1.8)	*0.495*
Rash	5	(2.5)	4	(2.8)	1	(1.8)	*0.675*
Pruritis	2	(1.0)	2	(1.4)	0	(0.0)	*0.371*
Hepatitis	21	(10.4)	16	(11.1)	5	(8.8)	*0.625*
Other	21	(10.4)	16	(11.1)	5	(8.8)	*0.625*

**presented percentages include only patients who received the therapy that is mentioned*

Supplemental table S2: Reasons for discontinuation of treatment (first line)

	All patients		65-74		75+		
	n	(%)	n	(%)	n	(%)	*p-value*
Reasons for stopping anti-PD							*0.026*
Planned	105	(15.4)	61	(18.2)	44	(12.7)	
Progression	270	(39.6)	149	(44.5)	121	(35.0)	
Toxicity	102	(15.0)	41	(12.2)	61	(17.6)	
Choice of patient	18	(2.6)	7	(2.1)	11	(3.2)	
Bad condition of patient	32	(4.7)	13	(3.9)	19	(5.5)	
Death	13	(1.9)	5	(1.5)	8	(2.3)	
Other	30	(4.4)	12	(3.6)	18	(5.2)	
Unknown	111	(16.3)	47	(14.0)	64	(18.5)	
Reasons for stopping ipilimumab							*<0.001*
Planned	123	(59.4)	78	(56.9)	45	(64.3)	
Progression	39	(18.8)	35	(25.5)	4	(5.7)	
Toxicity	29	(14.0)	20	(14.6)	9	(12.9)	
Bad condition of patient	9	(4.3)	2	(1.5)	7	(10.0)	
Death	4	(1.9)	1	(0.7)	3	(4.3)	
Other	2	(1.0)	1	(0.7)	1	(1.4)	
Unknown	1	(0.5)	0	(0.0)	1	(1.4)	
Reasons for stopping ipilimumab/nivolumab							*0.182*
Planned	15	(7.5)	12	(8.3)	3	(5.3)	
Progression	47	(23.4)	40	(27.8)	7	(12.3)	
Toxicity	75	(37.3)	51	(35.4)	24	(42.1)	
Patients' choice	3	(8.0)	2	(1.4)	1	(1.8)	
Bad condition of patient	16	(8.0)	9	(6.3)	7	(12.3)	
Death	16	(8.0)	9	(6.3)	7	(12.3)	
Other	4	(2.0)	2	(1.4)	2	(3.5)	
Unknown	25	(12.4)	19	(13.2)	6	(10.5)	

Supplemental table S3: Hospital admissions

	All patients		65-74		75+		
	N	(%)	N	(%)	N	(%)	*p-value*
Admitted (any time)	1233	(55.6)	668	(58.6)	565	(52.4)	*0.003*
Mean nr admissions (SE)	3.6	(0.22)	3.7	(0.23)	3.3	(0.22)	*0.263*
Reasons for admission*							
toxicity of treatment	1880	(24.2)	1077	(25.7)	803	(22.4)	*<0.001*
toxicity other treatment	138	(1.8)	111	(2.6)	27	(0.8)	
surgical complications	114	(1.5)	64	(1.5)	50	(1.4)	
palliative care	2312	(29.8)	1243	(29.7)	1069	(29.8)	
other	3316	(42.7)	1685	(40.2)	1631	(45.5)	
unknown	10	(0.1)	6	(0.1)	4	(0.1)	

**patients could be admitted more than once. Presented percentages represent the percentage of all admissions explained by the specified reason*

Supplemental table S4: Characteristics of patients discontinuing treatment due to toxicity

Anti-PD1

	Discontinuation due to toxicity		Other reason for discontinuation		Unknown or no discontinuation		*p-value*
Age	N	%	N	%	N	%	*0.006*
65-74	40	(41.2)	242	(53.5)	53	(40.2)	
75+	57	(58.8)	210	(46.5)	79	(59.8)	
Number of comorbidities							
0	11	(11.3)	46	(10.2)	14	(10.6)	*0.767*
1-2	39	(40.2)	199	(44.0)	63	(47.7)	
3 or more	43	(44.3)	197	(43.6)	50	(37.9)	
Unknown	4	(4.1)	10	(2.2)	5	(3.8)	
WHO classification							
0	47	(48.5)	222	(49.1)	68	(51.5)	*0.762*
1	33	(34.0)	160	(35.4)	42	(31.8)	
2	10	(10.3)	38	(8.4)	8	(6.1)	
3 or 4	0	(0.0)	6	(1.3)	2	(1.5)	
Unknown	7	(7.2)	26	(5.8)	12	(9.1)	
Response within 6 months							
No	286	(63.3)	42	(43.3)	67	(50.8)	*<0.001*
Yes	166	(36.7)	55	(56.7)	65	(49.2)	

Supplemental table S5: predictors of overall survival

	**Univariate**			**Multivariate**		
	HR	(95% C.I.)	*p-value*	HR	(95% C.I.)	*p-value*
Age						
65-74	Ref		*<0.001*	Ref		*0.002*
75+	1.22	(1.1-1.35)		1.18	(1.06-1.31)	
Sex						
Male	Ref		*0.510*	Ref		*0.718*
Female	0.97	(0.87-1.07)		0.98	(0.88-1.09)	
Number of comorbidities						
0	Ref		*0.001*	Ref		*0.315*
1-2	1.10	(0.94-1.29)		1.08	(0.92-1.27)	
3 or more	1.33	(1.13-1.56)		1.15	(0.98-1.35)	
Unknown	1.08	(0.73-1.61)		1.28	(0.86-1.91)	
WHO classification						
0	Ref		*<0.001*	Ref		*<0.001*
1	1.80	(1.58-2.04)		1.50	(1.32-1.71)	
2	2.99	(2.54-3.52)		2.23	(1.89-2.64)	
3 or 4	6.10	(4.90-7.59)		3.48	(2.77-4.37)	
Unknown	2.17	(1.85-2.56)		2.02	(1.70-2.39)	
**Tumour characteristics**						
Number of metastatic sites						
1-2	Ref		*<0.001*	Ref		*0.088*
3-5	1.15	(0.89-1.47)		1.24	(0.97-1.60)	
6 or more	2.27	(1.90-2.72)		2.17	(1.80-2.62)	
Unknown	1.28	(1.03-1.60)		1.29	(1.03-1.62)	
LDH						

Normal	Ref		*<0.001*	Ref		*<0.001*
Elevated	2.02	(1.81-2.24)		1.64	(1.47-1.83)	
Unknown	1.44	(1.19-1.74)		1.22	(1.00-1.49)	
Brain metastases						
No	Ref		*<0.001*	Ref		*<0.001*
Yes	1.79	(1.60-2.00)		1.56	(1.39-1.75)	
Unknown	1.72	(1.29-2.30)		1.63	(1.21-2.19)	
